# Hospital Memories and Six-Month Psychological Outcome: A Prospective Study in Critical Ill Patients with COVID-19 Respiratory Failure

**DOI:** 10.3390/jcm12093344

**Published:** 2023-05-08

**Authors:** Matteo Pozzi, Claudio Ripa, Valeria Meroni, Daniela Ferlicca, Alice Annoni, Marta Villa, Maria Grazia Strepparava, Emanuele Rezoagli, Simone Piva, Alberto Lucchini, Giacomo Bellani, Giuseppe Foti, the Monza Follow-Up Study Group

**Affiliations:** 1 Department of Emergency and Intensive Care, Fondazione IRCCS San Gerardo dei Tintori, 20900 Monza, Italy; mateo.pozzi@gmail.com (M.P.); alberto.lucchini@unimib.it (A.L.); giacomo.bellani@apss.tn.it (G.B.); giuseppe.foti@unimib.it (G.F.); 2 School of Medicine and Surgery, University of Milano-Bicocca, 20900 Monza, Italy; c.ripa1@campus.unimib.it (C.R.); mariagrazia.strepparava@unimib.it (M.G.S.); 3 Department of Medical and Surgical Specialties, Radiological Sciences and Public Health, University of Brescia, 25123 Brescia, Italy; simone.piva@unibs.it; 4 Department of Anesthesia, Critical Care and Emergency, Spedali Civili University Hospital, 25123 Brescia, Italy

**Keywords:** ARDS, PIC syndrome, ICU memories, COVID-19 pneumonia, long-term outcomes, psychological outcomes

## Abstract

ICU survivors suffer from various long-term physical and psychological impairments. Memories from the critical illness may influence long-term psychological outcome. In particular, the role of ICU memories in COVID-19 critically ill patients is unknown. In a prospective observational study, we aimed to investigate patients’ memories from the experience of critical illness and their association with a six-month psychological outcome involving quality of life evaluation. Patients’ memories were investigated with ICU Memory tool, while psychological outcome and quality of life were evaluated by means of a battery of validated questionnaires during an in-person interview at the follow-up clinic. 149 adult patients were enrolled. 60% retained memories from pre-ICU days spent on a general ward, while 70% reported memories from the in-ICU period. Delusional memories (i.e., memories of facts that never happened) were reported by 69% of patients. According to a multivariable analysis, the lack of pre-ICU memories was an independent predictor of worse psychological outcomes in terms of anxiety, depression and Post-traumatic Stress Disorder (PTDS). Factors associated with long-term outcome in ICU survivors are not still fully understood and patients’ experience during the day spent before ICU admission may be associated with psychological sequelae.

## 1. Introduction

The increased rate of ICU survival and the recent outbreak of the COVID-19 pandemic have created a growing number of ICU survivors [1,2]. Psychological problems and reduced health-related quality of life (HRQoL) are major concerns after ICU stay and can significantly burden critically ill patients for a long time [3]. In recent years, growing interest has been raised about these patient-centered conditions that, alongside and separately to mortality, have been advocated as important outcome measures in critical care [4]. 

ICU survivors, in particular after admission with acute respiratory distress syndrome (ARDS), have been described to be at increased risk of developing anxiety, depression, post-traumatic stress disorder (PTSD), fatigue, insomnia and reduced HRQoL, although the precise prevalence of these conditions is not known and several factors have been found to be associated with their development [5,6,7].

Among these, the role of patients’ memories from ICU stay on long-term psychological outcome has been already investigated. In particular, PTSD has been found to be associated with the number of adverse experiences remembered by patients [8], the presence of delusional memories (i.e., memories of facts that never happened) [9] and the absence of memories from the early period of critical illness [10]. 

Since a significant number of patients retain memories from the experience of critical illness, the identification of the association between their qualitative features and the development of adverse psychological sequelae could help to better understand the complex interplay between patients’ characteristics, illness severity and treatment exposure in determining long-term, patient-centered outcomes. 

The aim of this study is to investigate the association between memories from critical illness and the psychological long-term outcomes in a population of patients admitted to the ICU for COVID-19 related ARDS. 

## 2. Materials and Methods

Data were gathered into the context of a larger longitudinal observational follow-up study committed to the long-term evaluation of physical, cognitive and psychological outcome of critical-care patients (PICS Study) approved by local Ethical Committee (Comitato Etico Brianza—NP3369). The study was registered at clinicaltrial.gov (NCT: NCT04608994). Written informed consent was obtained from each participant at the time of the first follow-up visit.

### 2.1. Study Population

All consecutive adult (>18 years old) COVID-19 patients who received Mechanical Ventilation (MV) for at least 48 h and were discharged alive from ICU from 1 March 2020 to 30 March 2022 were included into the study (study flow chart shown in Figure S1). To avoid any influence of neuropsychological sequelae due to acute brain injury or pre-existing cognitive or memory deficit we excluded patients with cardiac arrest, stroke, intracranial bleeding, traumatic brain injury, pre-existing psychiatric disease and dementia. COVID-19 diagnosis was confirmed in all included patients by Polymerase Chain Reaction test.

### 2.2. Study Hospital

This prospective observational study was conducted in a large academic hospital (IRCCS San Gerardo dei Tintori, Monza), in Lombardy, Italy. Before the first Italian COVID-19 patient was recognized on 20 February 2020, the hospital had approximately 650 beds, including 30 adult ICU beds and 10 respiratory high-dependency unit beds. Being located in the epicenter of the first european COVID-19 pandemic wave, the huge burden of critically ill patients triggered a massive increase in ICU beds’ capacity, managing up to 90 mechanically ventilated patients simultaneously [1]. At the same time, several medical and surgical wards were converted into COVID-19 units. 

In order to face ICU beds’ shortage compared to the increasing amount of patients admitted to hospital for respiratory failure, we arranged a dedicated medical emergency team (MET) and a protocol was instituted for the use of helmet continuous positive airway pressure (CPAP) in patients who failed standard oxygen therapy [11,12]. Intravenous sedation was not used in general wards during CPAP. Patients were managed by ward teams (composed of non-ICU physicians and nurses from several disciplines) and daily screened by MET for possible ICU admission. 

### 2.3. Sedation Management

After ICU admission, a rapid sequence intubation administering Ketamine 1–1.5 mg/kg iv bolus, Midazolam 2–5 mg iv bolus and Rocuronium 1.2 mg/kg iv bolus was used for the maximization of respiratory support. During ICU stay, a standardized analgo-sedation practice in line with recent recommendations [13,14,15] was applied. During controlled mechanical ventilation a Richmond agitation sedation scale (RASS) −4/−5 was achieved administering iv sedation with propofol 4–5 mg/kg/h and remifentanil 0.05–0.5 mcg/kg/min. In the case of occurrence of severe hemodynamic instability, midazolam infusion was preferred to propofol, up to 0.2 mg/kg/h. On mechanical ventilation, all patients received a NMBA (Rocuronium 0.3–0.6 mg/kg/hr with daily interruption attempt). After switching to assisted mechanical ventilation, a RASS −1/0 was obtained by means titration of iv sedation with propofol or dexmedetomidine 0.2–1.4 mcg/kg/h and remifentanil 0.05–0.2 mcg/kg/min. In absence of contraindications (e.g., myocardial ischemia, agitation, evidence or suspicion of high intracranial pressure), sedative drugs were suspended on a daily basis for a sedation interruption trial which aimed to spare sedation, check neurological status and perform a spontaneous breathing trial, when indicated [16,17]. Serial assessments of sedation status were performed to adjust pharmacological interventions appropriately. After ventilatory weaning and oro-tracheal extubation, no benzodiazepines were administered, but RASS 0 was achieved via iv opiates and dexmedetomidine 0.2–0.6 mcg/kg/hr. In case of delirium persistent after pain control and non-pharmacological interventions, Haloperidol (0.5–1 mg i.v) was administered [18]. 

After September 2021, during the subsequent phases of pandemic emergency, critically ill COVID-19 patients were managed in a dedicated 12-bed ICU and high-dependency unit. 

### 2.4. Data Collection and Follow Up Measurement

Baseline characteristics (demographic, anthropometric, clinical severity data) and data regarding ICU treatment were collected at the time of ICU discharge. 

Institutional follow-up program provides longitudinal evaluation of all adult patients surviving the ICU by in-person evaluation. After ICU discharge, patients were scheduled for a visit six months later at the follow-up clinic. Those who denied their consent to participate or failed to be contacted were considered as lost at follow-up. 

A dedicated team, consisting of an ICU nurse and an ICU physician, provided a standard, multidimensional evaluation including the following measures:

1. Memories of hospital and ICU experience were evaluated by ICU memory tool (ICUMT) [9]. The questionnaire is composed of 8 questions about the presence of factual memories (i.e., recall of events that actually happened), memories of feeling (e.g., recall of anxiety, pain or fear) and delusional memories (i.e., recall of events that actually did not happen, such as nightmares and hallucinations) from ICU period (in-ICU memories). It also investigates the presence of recall from other periods of hospital stay, such as before (pre-ICU memories) and after ICU stay (post-ICU memories) and the presence of distress related to the re-experience of ICU memories (i.e., intrusive memories). The questionnaire has been proposed and validated in an italian version [19] and assesses the presence of memories of the three aforementioned domains by choosing them from a checklist of 8 items (see Supplementary Materials).
2. Psychological outcome was investigated by the administration of specific self-completed questionnaires:

- Hospital Anxiety Depression Scale (HADS), which provides a quantification of anxiety and depression using two subscores (HADS-A for anxiety and HADS-D for depression) that were considered as likely to be present with a threshold of 8 points for both subscore [20].
- Post-traumatic Checklist for DSM-5 (PCL-5) which investigates PTSD symptoms. A PCL-5 > 32 was considered consistent with the presence of PTSD [21].
- Insomnia Severity Index (ISI) which investigates sleep quality and is considered consistent with sleep disturbance for values > 8 [22].
- Fatigue Severity Score (FSS) which investigates the role of fatigue in activity limitation. An FSS > 36 was considered as an indicator of severe fatigue [23].

3. Health-related quality of life (HRQoL) was investigated by 36-item short-form health survey questionnaire), a validated questionnaire which covers different domains of patient-reported quality of life [24]. Items were scored as recommended [25] and two higher-order summary scores, the physical component summary (PCS) and the mental component summary (MCS) scores, were calculated [26]. For each item, a score above 50 was considered normal.

Primary outcome was the frequency of Anxiety, Depression, PTSD, sleep disturbance, Fatigue and reduced HRQoL according to the presence of retained memories from ICU or pre-ICU period. Secondary outcome was the frequency, among the patients who retained memories from ICU period, of the same conditions according to the presence of specific subtypes of memories. 

### 2.5. Statistical Analysis

Continuous variables were reported using median and interquartile range (IQR). Categorical variables were expressed as count and percentage. An a priori sample size calculation was not performed, since the study sample was represented by all eligible patients discharged during the study period who attended the follow-up clinic. 

Differences among continuous variables were tested by unpaired Student’s *t*-test or nonparametric Wilcoxon test according to the data distribution. 

To evaluate the association between the presence of memories and long-term outcomes, patients were initially divided according to the presence of pre-ICU or in-ICU memories. Pre-ICU memories from ICUMT were dichotomized as clear or null/blurred. Continuous and categorical variables were then confronted as before explained between these counterparts. Patients with ICU memories were then classified according to the quality of their recall. According to the ICUMT, three main groups of memories were identified: factual memories, feeling memories and delusional memories. In these groups, the distribution of the outcome scores and the frequency of the related conditions was compared as above specified. 

Finally, univariable logistic regression was used to examine the association between different psychological disorders (dependent variables) and different clinical confounders including ICU memories (independent variables). A set of clinically relevant variables (i.e., age, gender, SAPS-II and hospital length of stay) together with the presence of pre-ICU and in-ICU memories were tested into multivariable logistic regression models. Independent predictors of multivariable models were considered in the presence of a *p*-value < 0.05 (two-sided). No data imputation was provided for missing values. All tests were two-sided and a *p*-value less than 0.05 was considered statistically significant. Statistical analysis was performed with SPSS ver. 28 (SPSS Incorporation, Chicago, IL, USA).

## 3. Results

Among 271 patients successfully discharged from hospital during the study period, 149 attended the follow-up clinic and were included in the analysis (Figure S1). Table 1 resumes characteristics of the patients along with stratification according to the presence of in-ICU and pre-ICU memories. All patients but those intubated at the time of emergency department admission (N° = 32) were treated with CPAP at the ward before ICU admission. Median age was 60 (53–67) years and most patients were males (73%). More than half of patients had no more than one comorbidity (57%). Since the study period covered different subsequent pandemic waves, we reported in Table S1 and Figure S2 the distribution of patients enrolled according to these different time periods.

### 3.1. Pre-ICU and In-ICU Memories

**Figure 1:**
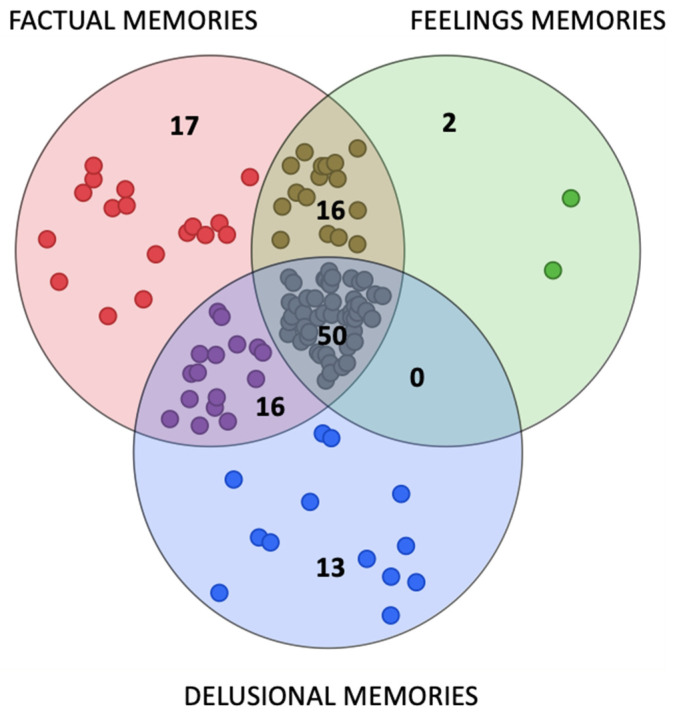
Occurrence of different types of in-ICU recalls in patients who retained ICU memories (n = 114). Each single dot corresponds to one single patient.

89/149 patients (60%) retained memories of pre-ICU hospital stay. On the contrary, 114/149 patients (77%) reported memories from the in-ICU period. Patients with pre-ICU or in-ICU memories were younger (*p* = 0.026 and *p* < 0.001, see Table 1) and had a longer pre-ICU length of stay (*p* = 0.034 and *p* = 0.029). Patients with ICU memories less frequently had multiple comorbidities (*p* = 0.01). 124/149 (82%) retained memories from at least one of these two phases. 25 patients (18%) had no memories of hospital stay at all. Among patients who retained ICU memories (N° = 114), the type of recall was analyzed (Figure 1 and Table S3). 99/114 patients (87%) retained factual memories. Of these, only 17 remembered factual memories (15% of the 114 patients with in-ICU memories). 68/114 patients (60%) retained feeling memories, of which only two retained only feeling memories. Delusional memories were recalled by 79/114 patients (69%). Of these, 13 (11% of the whole population) had only delusional memories. 50 (44%) patients recalled memories from all three areas. Frequency and co-occurrence of any type of recalls are depicted in Figure 1.

### 3.2. Anxiety, Depression, PTSD and Sleep Disturbance at Six Months from Discharge

30/149 (20%) patients were suffering from anxiety, and were more frequently treated with CRRT (10% vs. 2%, *p* = 0.022, see Table S4). Depression was present in 33/149 (22.1%) patients. Patients with depression were more frequently female (*p* = 0.017) and more frequently underwent CRRT (*p* = 0.001, see Table S5). PTSD was present in 11/149 (7%) patients, with no association to any patient characteristics (see Table S6). 28/149 (19%) patients suffered from sleep disturbance that was associated with younger age (*p* = 0.031, see Table S7). 

### 3.3. Fatigue and Health-Related Quality of Life 

Significant fatigue was reported in 51/149 (34%) of patients. Patients with fatigue were older (61 vs. 59 years old, *p* = 0.027), more frequently underwent tracheostomy (30% vs. 16%, *p* = 0.041, Table S8) and had longer in-hospital length of stay (46 vs. 39 days, *p* = 0.036, Table S8).

Health related quality of life was globally good. PCS was below threshold in 76/149 (51%) patients. Below-threshold PCS was associated with older age (*p* = 0.008), higher BMI (*p* = 0.042), longer hospital LOS (*p* = 0.017), prone position (*p* = 0.043) and treatment with corticosteroids (*p* = 0.021) (Table S9). MCS was below threshold in 121/149 (81%) patients, who were less frequently treated with ECMO (*p* = 0.044) (Table S10). 

### 3.4. Association between Pre-ICU Memories and Six Months Outcome 

The presence of pre-ICU memories was associated with a lower frequency of anxiety (33% vs. 67%, *p* = 0.001), depression (33% vs. 67%, *p* < 0.001) and fatigue (45% vs. 55%, *p* = 0.009) at six months. Consistently, patients with pre-ICU memories had lower HADS-A, HADS-D and FSS scores (*p* < 0.001). There was no association between the presence of pre-ICU memories and PTSD or sleep disturbance, although PCL-5 score was lower in these patients (*p* = 0.001). 

As regards quality of life, there was an association between pre-ICU memories and a lower frequency of below-threshold PCS (42% vs. 65%, *p* = 0.005). Consistently, both PCS and MCS were significantly higher in patients with pre-ICU memories (*p* = 0.001 and *p* < 0.001). Table 2 resumes results of HADS, PLC-5, ISI, FSS and SF-36 questionnaire along with stratification for the presence of pre-ICU and in-ICU memories. Detailed results of SF-36 questionnaire are provided in Supplementary Materials (Table S11). 

### 3.5. Association between In-ICU Memories and Psychological Outcome

No association was found between the presence of recalls from ICU experience and the presence of anxiety, depression, PTSD and sleep disturbance. Nevertheless, patients with ICU memories had lower PCL-5 scores (*p* = 0.037) and less frequently experienced fatigue (30% vs. 49%, *p* = 0.041) (Table 2). Among patients with in-ICU memories, both below-threshold PCS and MCS were significantly less frequent (*p* = 0.006 and *p* = 0.024). PCS was higher in patients retaining ICU memories (*p* = 0.006).

### 3.6. Multivariable Analysis

In Table 3 are summarized the results of the multivariable analysis. The presence of pre-ICU memories was constantly retained as an independent predictor of lower frequency of anxiety, depression, and PTSD. The hospital length of stay was independently associated with the development of anxiety and lower PCS score. Female gender was an independent predictor for development of depression. 

## 4. Discussion

In this work, we aimed to investigate the association between patients’ memories from critical illness due to COVID-19-related respiratory failure and a multidimensional six-month psychological outcome, which involved a quality of life evaluation. The main findings of our work can be summarized as follows:

First, most patients retained memories from ICU stay, and a similar percentage had memories of the hospital days before ICU admission. Less than one out five patients did not have any memories from any time of their hospital experience. Among those retaining ICU memories, the number of patients with only delusional memories was very small (11%). Moreover, the frequency of adverse psychological outcomes was comparable to previous studies [3,27].

Second, the absence of memories from the period before ICU admission was independently associated with the presence of anxiety, depression and fatigue. The absence of in-ICU memories was independently associated with a below-threshold mental component of HRQoL, while absence of post-ICU memories was an independent predictor of a below-threshold physical component of HRQoL. 

Third, as regards the qualitative characteristics of ICU recall, delusional memories were not independently associated with any psychological outcome, while the presence of feeling memories was an independent predictor of PTSD. 

After ICU discharge, all the patients are overwhelmed by a burden of re-adaptation to normal life and returning to pre-admission activities. Often compromised by psychological sequelae and physical impairments, this transition period could be crucial and may end in an almost full recovery or in an unsatisfactory re-appropriation of daily-life abilities [28].

Many studies found that ICU survivors are at higher risk for the development of long-term adverse psychological outcomes, such as anxiety, depression, PTSD and ultimately reduced quality of life, and there are no proven strategies able to mitigate long-term sequelae and improve patients outcome [29]. Moreover, the ability to predict the development of long-term physical, cognitive and mental impairment is crucial to address high-risk patients to accurate screening and to personalized post-discharge care. Risk factors for the development of adverse psychological sequelae after critical illness are not fully understood, albeit many patient and illness-related variables have been proposed. The issue has been recently addressed by a consensus document [7], which found that symptoms of depression, traumatic memories, lack of social support and older age were associated with the development of psychological problems.

Previous works suggested the role of patients’ memories from hospital experience as a risk factor for developing adverse mental outcomes. In particular, the absence of recall from ICU [30] and the presence of the so-called “delusional memories” (i.e., memories of facts or circumstances that actually never happened) [9,31,32] or other negative feeling memories [33] were found to be associated with the development of PTSD and psychiatric symptoms and reduced quality of life. On the contrary, the presence of factual memories seemed to protect ICU survivors from adverse psychological sequelae [30]. 

The prevalence of anxiety, depression and PTSD was relatively low (respectively, 20%, 22% and 7%) in our population and comparable to previous cohort studies on COVID-19 ICU survivors [34]. Similarly, quality of life at six months was comparable to previous findings, with a notably high prevalence of low PCS and low MCS (namely 51% and 81% of patients), in spite of insomnia experienced by 19% and fatigue complained of by 34% of patients.

Most patients (77%) retained memories from the ICU. Of these, a large proportion (87%) had factual memories, and in particular recalled light and medical staff. Memories of feelings were retained by a smaller number of patients (60%), and most of them recall anxiety. A similar proportion of patients had delusional memories principally recalling dreams, hallucinations or nightmares. In most patients, two or three types of recall are simultaneously present (Figure 1) and only 13/114 (11%) patients had only delusional memories. Pre-ICU memories are reported by a similar percentage of patients (60%). Previous work reported a similar rate of survivors recalling ICU experience [33,35,36,37], even if differences in study design may limit generalizability of results. In particular, it can be speculated that specific populations (e.g., trauma patients) [38] could differ from ARDS patients for many factors affecting memory retention. Similarly, time of questionnaire administration (e.g., immediately after discharge vs. at 6 months) may affect the rate of recall and the degree of processing of the ICU experiences. 

It can be speculated that COVID-19 patients, and in particular in the early, dramatic, phases of pandemic emergency, could be exposed to a very stressful experience [39,40]. Patients lived in a high degree of social isolation, suffered from a mostly unknown illness and were cared for by healthcare providers wearing personal protective equipment. In spite of this, the rate of PTSD was low in our population, consistent with previous and recent works accounting for the absence of a significant increase of psychological impairment in COVID-19 in comparison to non-COVID critically ill survivors [27,41,42]. 

Frequency of anxiety and low PCS was significantly different in patients enrolled in different pandemic waves. It can be hypothesized that the evolution of the emergency situation and healthcare system overload could have attenuated ambiental factors able to influence long-term outcome. This hypothesis needs confirmation in specifically addressed studies. 

Early amnesia for the very first phase of critical illness (i.e., the time spent in hospital before ICU admission) was independently associated with the presence of anxiety, depression and fatigue. On the contrary, specific characteristics of ICU recalls, and in particular the presence of delusional or factual memories were not independently associated with long-term psychological outcome. Both delusional and factual memories have been found to be associated with psychological outcomes. 

Different studies reported the possible protective role of factual memories against the development of PTSD and reduced quality of life after ICU discharge. In this way, a better re-elaboration of a traumatic experience relying on memories undressed from the emotional burden could help the patients achieve a better psychological condition [9] and, ultimately, a better quality of life [30]. 

The discrepancy between these results and our findings could be explained by different factors. First, in the study from Jones et al., ICU memories were collected very early after ICU discharge (two weeks), while we investigated ICU memories and patients’ outcome at six months. Different types of memories may have different persistence over time and different study designs could impact the ability to collect certain types of recall, regardless of their specific role in outcome determination. 

Moreover, the percentage of patients with only delusional memories was higher in previous studies than in the present work. In addition to the already-mentioned time-related factor, different circumstances may account for this discrepancy. Above others, the role of diagnosis at ICU-admission cannot be ruled out, as COVID-19 patients may significantly differ from other types of ICU populations. This issue needs to be addressed by further comparative investigations. 

In our work, the absence of memories from index critical illness was associated with anxiety, depression, fatigue and reduced quality of life. A previous study [10] reported a similar association between PTSD development and memories from the ICU period, but this is, to the best of our knowledge, the first report highlighting the importance of hospital experience before ICU admission. 

This so-called “early amnesia” phenomenon could be interpreted as a proxy of patients’ severity, as this phase (i.e., the time spent in hospital before ICU admission) usually corresponds to the peak of clinical and physiological derangement that culminates in treatment upgrade. In this way, more severely ill patients could be burdened by a more severe brain dysfunction and, ultimately, would less frequently retain memories. In addition to this, the role of delirium in this phase cannot be ruled out. Differently from previous studies, “early amnesic” patients were slightly older (61 vs. 58 years old) and spent a shorter period on the general ward (two vs. four days) before ICU admission, and it can be speculated that both these circumstances could be associated with a higher illness severity. On the other hand, it is worth noting as all of them underwent the same, protocolized medical and ventilatory treatment (i.e., helmet CPAP) and there were no further differences in other patient- or treatment-related variables. 

Worthy of mention is that neuro-psychological symptoms seem to affect some COVID-19 ARDS survivors, independently from the respiratory failure [43]. This suggests a direct brain involvement in SARS-CoV-2 infection [44,45]. In particular, micro-thrombotic events and inflammation have been proposed as possible mechanisms. Consequently, depression, anxiety and other psychological outcomes could last beyond the resolution of respiratory failure [46]. Despite these findings, biochemical data were not collected in this study, and no suggestions could be developed.

Taken together, our results seem to suggest the possible independent role of patients’ experience while admitted on a general ward when healthcare is provided before ICU admission. As ICU amnesia may do [10], absence of pre-ICU memories could disorient patients and cause a break in the chain of events that brought them from home to ICU, increasing the prevalence of anxiety, depression and PTSD. Further studies are needed to clarify this hypothesis. 

Specific intervention, such as patient diaries, has been proposed to address this impairment in recall ability from ICU experience, with the aim to improve psychological outcome. Our result, for the first time, highlights the possibility that hospital days spent before ICU admission might stem factors able to influence long-term ICU outcome and that could be possibly addressed by specific interventions aimed to improve patients’ experiences.

The main strengths of this study are the large population of COVID-19 ICU survivors and the long follow-up period at which outcome is measured. 

The clinical homogeneity of the population may limit the generalizability of the study, but also, in the setting of protocolized treatment bundles, reduces the possibility that our results could be biased by unrecognized factors related to patients’ management or diagnosis. 

### Study Limitation

This study has several limitations that must be acknowledged. Although we report an independent association between pre-ICU amnesia and psychological distress, our observational design prevents us from assessing any causative mechanism. As it has been suggested that anxiety, depression and PTSD may negatively influence the ability to report memories [47], it can be speculated that some patients may report amnesia from critical illness because of pre-existing anxiety and depression. The exclusion of patients with a pre-admission diagnosis of psychiatric disease may have reduced the risk of such a bias, even if it cannot be ruled out ultimately.

On the other side, the risk of a recall bias must be ascertained. Patients suffering from psychological distress could be more prone to thoroughly search one’s memory for a cause of their problems, and in particular patients with less factual memories could be more prone to fill the void with illusive and erroneous recalls. This circumstance deserves two considerations. First, the ICU memory tool does not allow us to collect delusive memories from the period prior to ICU admission, and so prevented us from recognizing any association between delusional memories coming from general ward experience and long-term psychological outcome. Secondly, it is worth noting that patients who lack memories from the pre-ICU period and those retain them did not differ in terms of delusional memories from ICU stay.

We did not collect data about sedation strategy and occurrence of delirium. Both these factors have been reported as associated with PTSD development [48,49] and with retained memories from the ICU [50,51]. ICU patients may suffer from higher levels of PTSD, as their ability to process information during the exposure to the stressor situation is compromised by several factors, such as clinical severity, brain dysfunction, sedative drugs and delirium. Our work cannot investigate the role of these specific conditions in psychological distress generation and the possible interplay between memories, brain dysfunction, and psychological outcome, and our results must be considered in light of this major limitation. Further studies are needed to specifically address this topic.

Finally, we were not able to evaluate all discharged patients since 122 out of 271 discharged patients (45%) did not attend the follow up (see Figure S1). This was mainly due to restriction of hospital access for outpatients’ evaluation and the unwillingness of patients to attend the follow-up clinic during the subsequent pandemic waves which burdened our country. This may have introduced an obvious selection bias that limits the generalizability of our results.

## 5. Conclusions

In a prospective observational cohort study including critically ill patients admitted to ICU for COVID-19 respiratory failure and followed up over a prolonged time of investigation, we reported that long-term psychological outcome and quality of life were independently associated with the presence of memories from hospital experience, in particular from the period before ICU admission. We also reported that feeling memories, but not delusional of factual memories, were associated with different patient outcomes.

## Figures and Tables

**Table 1:** Patients’ demographic and clinical characteristics according to the presence of pre-ICU (i.e., memories of hospital stay before ICU admission) or in-ICU memories (i.e., memories of the period spent in ICU).

	All Patients	Pre-ICU Memory	No Pre-ICU Memory	*p*	In-ICU Memory	No in-ICU Memory	*p*
	(N° = 149)	(N° = 89)	(N° = 60)		(N° = 114)	(N° = 35)	
**Age, years,** **median (IQR)**	60 (53–67)	58 (51–65)	61 (57–68)	**0.026**	58 (51–65)	66 (59–69)	**<0.001**
**Sex, M, N° (%)**	109 (73)	67 (76)	42 (70)	0.405	86 (76)	27 (66)	0.223
**BMI, kg/m^2^,** **median (IQR)**	29.1 (26.6–32.8)	29 (26.6–32.4)	29.4 (26.8–33.9)	0.334	29.1 (26.7–33.1)	28.7 (26.6–32.5)	0.720
**Charlson Index,** **median (IQR)**	0 (0–0)	0 (0–0)	0 (0–1)	0.283	0 (0–0)	0 (0–0)	0.150
**N of comorbidities,** **n (%)**							
**0**	41 (28)	21 (24)	20 (34)	0.198	34 (30)	7 (20)	**0.010**
**1**	44 (30)	29 (33)	15 (25)		34 (30)	10 (29)	
**2**	25 (17)	19 (22)	6 (10)		23 (21)	2 (6)	
**3**	17 (12)	8 (9)	9 (15)		11 (10)	6 (17)	
**4**	21 (14)	11 (13)	9 (15)		10 (9)	10 (29)	
**SAPS II, median (IQR)**	35 (29–43)	35 (28–42)	37 (29–44)	0.327	35 (29–43)	39 (30–44)	0.464
**General ward pre-ICU, Length of stay, days, median (IQR)**	3 (1–5)	4 (1–6)	2 (0–4)	**0.034**	3 (1–5)	1 (0–5)	**0.029**
**ICU length of stay, days, median (IQR)**	18 (11–28)	16 (9–26)	18 (13–31)	0.150	16 (10–27)	19 (13–28)	0.151
**Hospital length of stay, days,** **median (IQR)**	41 (29–52)	38 (29–49)	44 (32–59)	0.075	39 (29–49)	45 (32–59)	0.090
**MV duration, days,** **median (IQR)**	13 (9–21)	12 (6–18)	13 (9–22)	0.341	13 (7–20)	13 (8–21)	0.897
**Tracheostomy, n (%)**	30 (20)	15 (17)	15 (25)	0.230	23 (21)	7 (20)	0.927
**ECMO, n (%)**	16 (11)	10 (11)	6 (10)	0.793	15 (13)	1 (3)	0.083
**Pronation, n (%)**	119 (80)	69 (78)	50 (83)	0.459	91 (81)	28 (80)	0.945
**Corticosteroids, n (%)**	100 (68)	61 (69)	40 (68)	0.706	73 (65)	28 (80)	0.099
**CRRT, n (%)**	5 (3)	2 (2)	3 (5)	0.364	4 (4)	1 (3)	0.832
**Vasopressors, n (%)**	24 (16)	14 (16)	10 (17)	0.891	17 (15)	7 (20)	0.514

BMI: body mass index; CRRT: continuous renal replacement therapy; ECMO; extracorporeal membrane oxygenation; ICU: intensive care unit; MV: mechanical ventilation; SAPS II: simplified acute physiological score II.

**Table 2:** HADS-A, HADS-D, PCL-5, ISI and FSS, PCS, MCS scores in the whole population and according to the presence of pre-ICU (i.e., memories of hospital stay before ICU admission) or in-ICU memories (i.e., memories of the period spent in ICU).

	All Patients (N° = 149)	Pre-ICU Memories (N° = 89)	No Pre-ICU Memories (N° = 60)	*p*	In-ICU Memories (N° = 114)	No In-ICU Memories (N° = 35)	*p*
**Anxiety and depression**							
**HADS-A, median (IQR)**	3 (1–7)	2 (1–6)	5 (2–8)	**0.001**	3 (1–7)	4 (1–7)	0.118
**HADS-A > 8, N° (%)**	30 (20)	10 (11)	20 (33)	**0.001**	22 (19)	8 (23)	0.646
**HADS-D, median (IQR)**	3 (1–7)	2 (1–5)	6 (2–9)	**<0.001**	3 (1–6)	4 (2–8)	0.106
**HADS-D > 8, N° (%)**	33 (22)	11 (12)	22 (37)	**<0.001**	22 (19)	11 (31)	0.131
**Post-traumatic stress disorder**							
**PCL-5, median (IQR)**	6 (1–14)	4 (1–10)	9 (5–19)	**0.001**	5 (1–11)	11 (3–17)	**0.037**
**PCL-5 > 32, N° (%)**	11 (7)	4 (5)	7 (12)	0.101	10 (9)	1 (3)	0.242
**Sleep disturbance**							
**ISI, median (IQR)**	3 (1–6)	3 (1–5)	3 (2–7)	0.287	3 (1–6)	3 (2–5)	0.815
**ISI > 8, N° (%)**	28 (19)	16 (18)	12 (20)	0.757	24 (21)	4 (11)	0.202
**Fatigue**							
**FSS, median (IQR)**	25 (15–42)	21 (12–36)	31 (19–49)	**<0.001**	22 (13–39)	35 (20–48)	**0.006**
**FSS > 36, N° (%)**	51 (34)	23 (26)	28 (47)	**0.009**	34 (30)	17 (49)	**0.041**
**Health-related quality of life**							
**PCS, median (IQR)**	48.97 (37.07–56.97)	52.72 (40.90–57.90)	44.77 (33.51–54.90)	**0.001**	51.73 (38.86–57.40)	39.40 (33.7–51.58)	**0.006**
**PCS < 50, N° (%)**	76 (51)	37 (42)	39 (65)	**0.005**	51 (45)	25 (71)	**0.006**
**MCS, median (IQR)**	39.88 (25.41–48.74)	45.53 (35.10–49.23)	27.07 (17.63–4503)	**<0.001**	40.91 (27.15–49.11)	37.29 (22.42–47.43)	0.158
**MCS < 50, N° (%)**	121 (81)	68 (76)	53 (88)	0.068	88 (77)	33 (94)	**0.024**

FSS: fatigue severity scale; HADS-A: hospital anxiety and depression scale—anxiety; HADS-D: hospital anxiety and depression scale—depression; ISI: insomnia severity index; MCS: mental component summary measures of quality of life; PCL-5: PTSD checklist for DSM-5; PCS: physical component summary measures of quality of life.

**Table 3:** Multivariable analysis.

**Anxiety (HADS-A > 7)**	**OR**	**CI 95%**	***p***
Age (each year)	0.970	0.924–1.017	0.202
Gender (male)	0.482	0.179–1.303	0.154
SAPS-II	1.034	0.993–1.077	0.101
**Hospital length of stay (days)**	**1.023**	**1.003–1.043**	**0.020**
Presence of in-ICU memories	1.878	0.575–6.128	0.285
**Presence of pre-ICU memories**	**0.240**	**0.089–0.642**	**0.004**
**Depression (HADS-D > 7)**	**OR**	**CI 95%**	***p***
Age (each year)	0.970	0.928–1.014	0.190
**Gender (male)**	**0.392**	**0.155–0.988**	**0.049**
SAPS-II	1.018	0.980–1.057	0.350
Hospital length of stay (days)	1.017	0.998–1.035	0.080
Presence of in-ICU memories	0.737	0.262–2.075	0.565
**Presence of pre-ICU memories**	**0.242**	**0.096–0.612**	**0.003**
**PTSD (PCL-5 > 32)**	**OR**	**CI 95%**	***p***
Age (each year)	0.957	0.898–1.021	0.202
Gender (male)	0.424	0.112–1.607	0.214
SAPS-II	1.024	0.969–1.082	0.403
Hospital length of stay (days)	0.995	0.962–1.029	0.770
Presence of in-ICU memories	4.690	0.513–43.013	0.116
**Presence of pre-ICU memories**	**0.199**	**0.049–0.815**	**0.025**
**Insomnia (ISI > 8)**	**OR**	**CI 95%**	***p***
Age (each year)	0.974	0.932–1.018	0.765
Gender (male)	0.620	0.238–1.616	0.243
SAPS-II	1.016	0.978–1.055	0.420
Hospital length of stay (days)	0.998	0.977–1.019	0.840
Presence of In-ICU memories	2.849	0.714–11.361	0.112
Presence of pre-ICU memories	0.606	0.236–1.557	0.301
**Fatigue (FSS > 36)**	**OR**	**CI 95%**	***p***
Age (each year)	1.028	0.986–1.070	0.195
Gender (male)	1.425	0.598–3.398	0.418
SAPS-II	1.003	0.971–1.038	0.832
Hospital length of stay (days)	1.013	0.996–1.030	0.119
Presence of in-ICU memories	0.620	0.250–1.539	0.304
Presence of pre-ICU memories	0.528	0.243–1.149	0.108
**Low PCS (PCS < 50)**	**OR**	**CI 95%**	***p***
Age (each year)	1.028	0.990–1.067	0.137
Gender (male)	0.818	0.360–1.856	0.631
SAPS-II	0.999	0.968–1.031	0.957
**Hospital length of stay (days)**	**1.020**	**1.002–1.039**	**0.021**
Presence of in-ICU memories	0.510	0.196–1.326	0.162
Presence of pre-ICU memories	0.534	0.247–1.153	0.110
**Low MCS (MCS < 50)**	**OR**	**CI 95%**	***p***
Age (each year)	0.996	0.953–1.041	0.872
Gender (male)	1.875	0.707–4.969	0.212
SAPS-II	0.986	0.950–1.023	0.463
Hospital length of stay (days)	0.993	0.973–1.013	0.517
Presence of in-ICU memories	0.217	0.044–1.081	0.062
Presence of pre-ICU memories	0.587	0.215–1.602	0.288

SAPS-II: Sequential acute physiological score II.

## Data Availability

The data presented in this study are available on request from the corresponding author.

## Supplementary Materials

The following supporting information can be downloaded at: https://www.mdpi.com/article/10.3390/jcm12093344/s1. Figure S1: Study flow chart. Figure S2: Distribution of enrolled patients according to the different consecutive pandemic waves. Table S1: Distribution of enrolled patients according to the different consecutive pandemic waves. Table S2: HADS-A, HADS-D, PCL-5, ISI, FSS, PCS and MCS scores and corresponding prevalence of anxiety, depression, PTSD, sleep disturbance, Fatigue and Reduced HRQoL among enrolled patients in each consecutive pandemic wave.
