# Supplementary material for: Hospital Memories and Six-Month Psychological Outcome: A Prospective Study in Critical Ill Patients with COVID-19 Respiratory Failure
Source: J Clin Med. 2023 May 8;12(9):3344. doi: 10.3390/jcm12093344 (PMC10179721; doi:10.3390/jcm12093344)
Supplement: Supplementary file 1 [file jcm-12-03344-s001.zip › jcm-2341099-supplementary.pdf]

Hospital memories and six-months psychological outcome:

a prospective study in critical care COVID-19 patients.

Matteo Pozzi [1], Claudio Ripa [2], Valeria Meroni [1], Daniela Ferlicca [1], Alice Annoni [1], Marta Villa [1], Simone Piva [3,4], Maria Grazia Strepparava [2], Emanuele Rezoagli [1,2], Alberto Lucchini [1,2], Giacomo Bellani [1.2], Giuseppe Foti [1.2] and the Monza Follow-Up Study Group [†]

[1] Department of Emergency and Intensive Care, ASST Monza, 20900 Monza, Italy
[2] School of Medicine and Surgery, University of Milano-Bicocca, 20900 Monza, Italy
[3] Department of Medical and Surgical Specialties, Radiological Sciences and Public Health, University of Brescia, Brescia, Italy
[4] Department of Anesthesia, Critical Care and Emergency, Spedali Civili University Hospital, Brescia, Italy
[†] **Monza Follow-Up Study Group Collaborators**: SSA, Francesca Bettini, Diego Boaretto, Alfio Bronco, Mariangela Calabria, Gianmarco Carenini, Mara Clementi, Stefano Gatti, Fabrizia Mauri, Melissa Racis, Simone Sosio, Alessandra Valentino, Veronica Vigo.

Supplementary Material

**Supplementary Materials**	pag.
**ICU memory tool**	2
**Figure S1.** Study Flow Chart.	3
**Figure S2.** Distribution of enrolled patients according to the different consecutive pandemic waves**.**	4
**Table S1.** Distribution of enrolled patients according to the different consecutive pandemic waves**..**	5
**Table S2.** HADS-A, HADS-D, PCL-5, ISI, FSS, PCS and MCS scores and corresponding prevalence of anxiety, depression, PTSD, sleep disturbance, Fatigue and Reduced HRQoL among enrolled patients in each consecutive pandemic wave.	6
**Table S3**. Types of ICU memories for patients who retained ICU memories (n = 114).	8
**Table S4.** Patients characteristic according to the presence of Anxiety at six months from discharge.	9
**Table S5.** Patients characteristic according to the presence of Depression at six months from discharge.	10
**Table S6.** Patients' characteristics according to the presence of Post Traumatic Stress Disorder (PTSD).	11
**Table S7**. Patients' characteristics according to the presence of Insomnia.	12
**Table S8**. Patients' characteristics according to the presence of Fatigue.	13
**Table S9.** Patients' characteristic according to the presence of below-threshold Physical Component Score (PCS) of Short Form Health Survey 36 (SF-36) at six months from discharge.	14
**Table S10.** Patients' characteristics according to the presence of below-threshold Mental Component Score (MCS) of Short Form Health Survey 36 (SF-36) at six months from discharge.	15
**Table S11**. Short Form Health Survey 36 (SF-36) results according to the presence of pre-ICU and ICU memories.	16

ICU Memory Tool.

1. Do you remember being admitted to hospital?
   - ☐ Clearly
   - ☐ Hazily
   - ☐ Not at all

2. Can you remember the time in hospital before you were admitted to intensive care?
   - ☐ All of it
   - ☐ Some of it
   - ☐ Nothing

3. Do you remember being in intensive care?
   - ☐ Yes
   - ☐ No

4a. Do you remember all the stay clearly?
   - ☐ Yes
   - ☐ No

4b. What do you remember? (circle those things you remember)

☐ Family ☐ Alarms ☐ Voices ☐ Lights ☐ Faces ☐ Suctioning ☐ Clock ☐ Darkness

☐ Breathing tube ☐ Tube in your nose ☐ Ward round ☐ Being uncomfortable

☐ Panic ☐ Pain ☐ Feeling confused ☐ Feeling down ☐ Feeling anxious/frightened

☐ Hallucinations ☐ Nightmares ☐ Dreams ☐ Feeling that people were trying to hurt you

4c. If you had any feelings that someone was trying to hurt or harm you while you were in intensive care can you please describe these feelings below

…………………………………………………………………………………………[free text]

4d. If you had nightmares or hallucinations while you were in intensive care could you please describe these:

…………………………………………………………………………………………[free text]

5. Do you remember being transferred from intensive care to the general wards?
   - ☐ Clearly
   - ☐ Hazily
   - ☐ Not at all

6. Have you had any unexplained feelings of panic or apprehension?
   - ☐ Yes
   - ☐ No

6a. If yes: What were you doing when these feelings happened?

…………………………………………………………………………………………[free text]

7. Have you had any intrusive memories from your time in hospital or of the event that lead up to your admission?
   - ☐ Yes
   - ☐ No

7a. If yes to 7: What were you doing when these intrusive memories happened?

…………………………………………………………………………………………[free text]

7b. If yes to 7: What did these memories consist of (e.g. frightening nightmares)?

8. Have you talked about what happened to you in intensive care with:-
   - ☐ A member of your family
   - ☐ A nurse on the ward
   - ☐ A friend
   - ☐ A doctor on the ward
   - ☐ Your family doctor

Figure S1. Study Flow Chart.

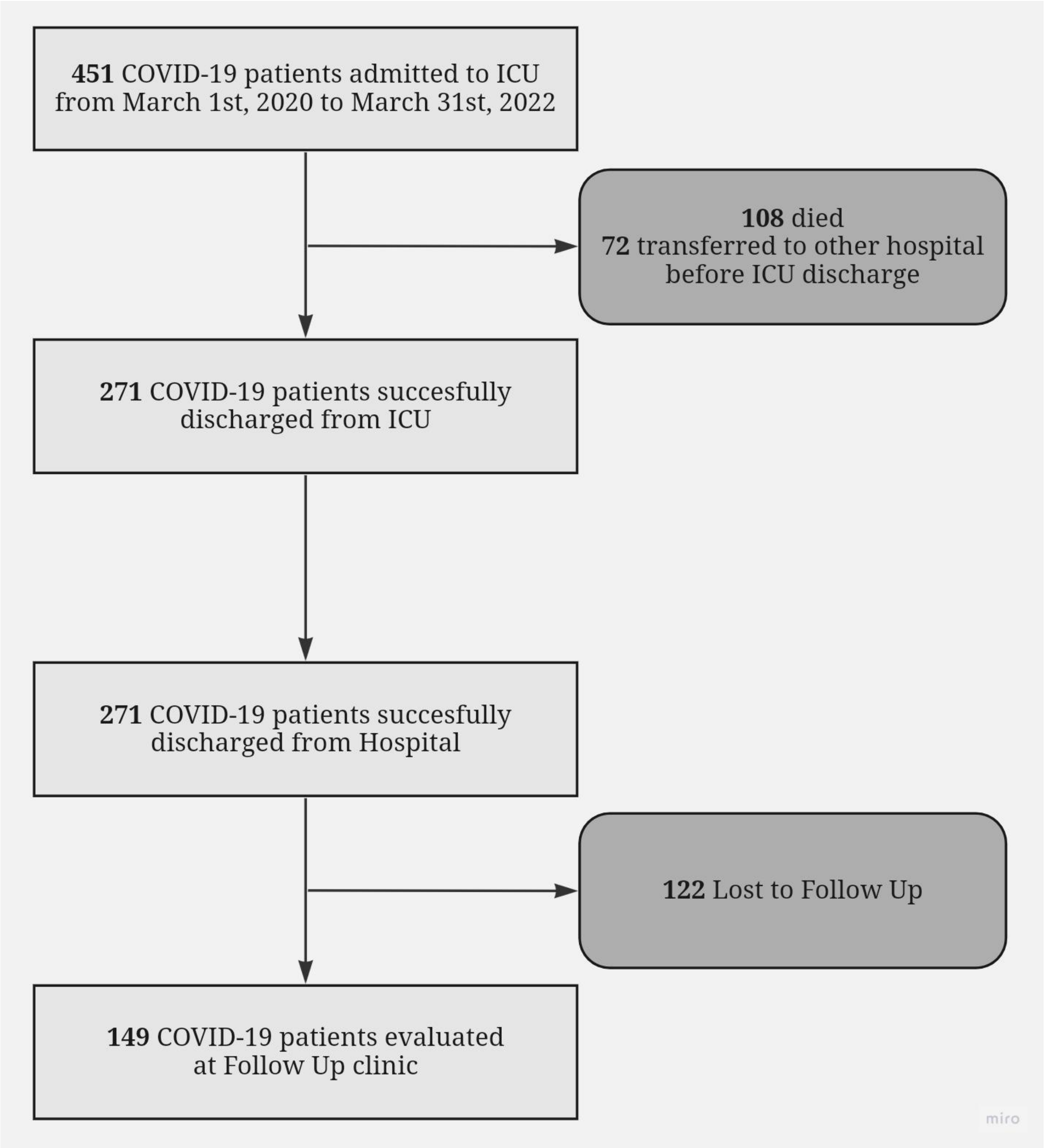

Figure S2. Distribution of enrolled patients according to the different consecutive pandemic waves**:** 1st pandemic wave (from March 2020 to September 2020), 2nd pandemic wave (from October 2020 to January 2021), 3rd pandemic wave (from February 2021 to September 2021), 4th pandemic wave (from October 2021 to March 2022).

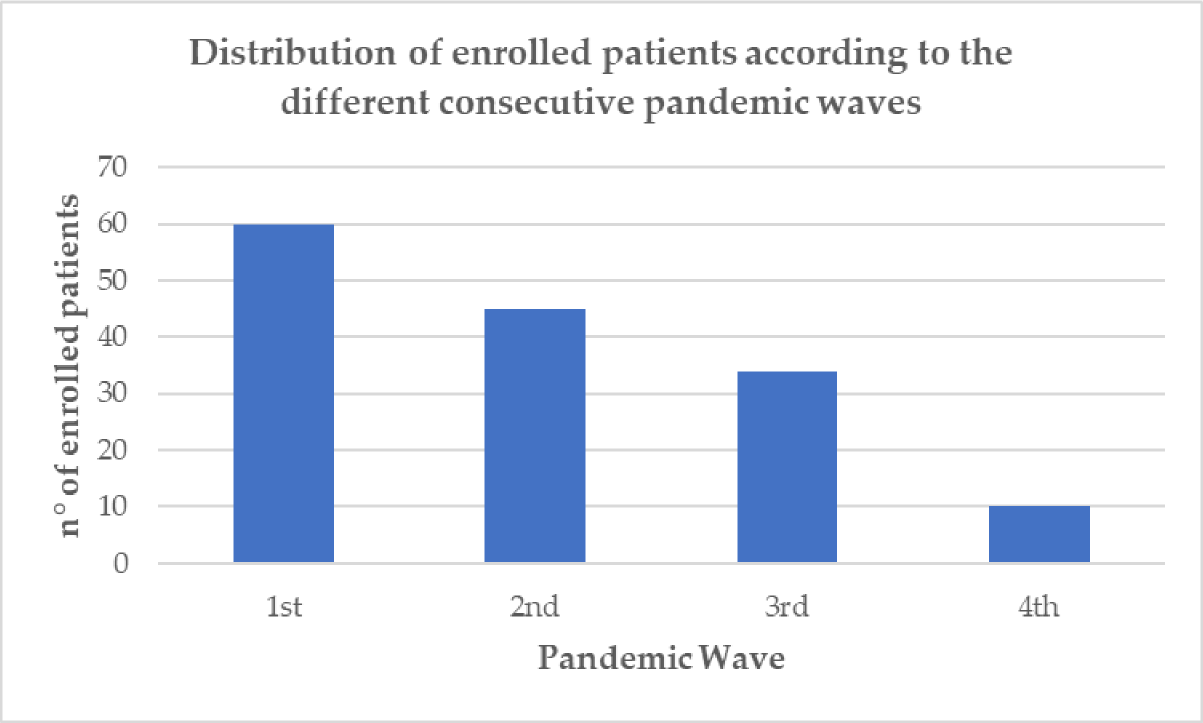

Table S1. Distribution of enrolled patients according to the different consecutive pandemic waves**.** 1st pandemic wave (from March 2020 to September 2020), 2nd pandemic wave (from October 2020 to January 2021), 3rd pandemic wave (from February 2021 to September 2021), 4th pandemic wave (from October 2021 to March 2022).

Pandemic Wave	Month	N° of enrolled patients	TOT enrolled patients
**1st**	03/2020	41	60
	04/2020	17	
	05/2020	0	
	06/2020	0	
	07/2020	0	
	08/2020	0	
	09/2020	2	
**2nd**	10/2020	12	45
	11/2020	23	
	12/2020	4	
	01/2021	6	
**3rd**	02/2021	13	34
	03/2021	11	
	04/2021	6	
	05/2021	1	
	06/2021	1	
	07/2021	1	
	08/2021	0	
	09/2021	1	
**4th**	10/2021	3	10
	11/2021	0	
	12/2021	5	
	01/2022	1	
	02/2022	0	

	03/2022	1	
**TOT**		149	**149**

Table S2. HADS-A, HADS-D, PCL-5, ISI, FSS, PCS and MCS scores and corresponding prevalence of anxiety, depression, PTSD, sleep disturbance, Fatigue and Reduced HRQoL among enrolled patients in each consecutive pandemic wave: 1st pandemic wave (from March 2020 to September 2020), 2nd pandemic wave (from October 2020 to January 2021), 3rd pandemic wave (from February 2021 to September 2021), 4th pandemic wave (from October 2021 to March 2022). p-value (two-tailed) examines the association between specific scores or conditions (dependent variables) with different pandemic waves (independent variable) by univariate logistic (for categorical variables) or linear (for continuous variables) regression models.

	**1st wave (n = 60)**	**2nd wave (n = 45)**	**3rd wave (n = 34)**	**4th wave (n = 10)**	**p**
**Anxiety**					
**HADS-A, median (IQR)**	3 (1 - 6)	4 (1 - 8)	3 (1 - 7)	2 (2 - 5)	0.421
**HADS-A > 8, N° (%)**	8 (13)	14 (31)	8 (24)	0 (0)	**0.022**
**Depression**					
**HADS D, median (IQR)**	3 (1 - 7)	3 (1 - 7)	4 (1 - 8)	3 (2 - 5)	0.692
**HADS-D > 8, N° (%)**	13 (22)	10 (22)	9 (26)	1 (10)	0.710
**Post-traumatic Stress Disorder**					
**PCL-5, median (IQR)**	5 (1 - 10)	8 (1 - 16)	10 (2 - 17)	2 (1 - 6)	0.070
**PCL > 32, N° (%)**	3 (5)	3 (7)	5 (15)	0 (0)	0.233
**Sleep Disorder**					
**ISI, median (IQR)**	3 (2 - 6)	2 (1 - 9)	3 (1 - 5)	2 (1 - 3)	0.468
**ISI > 8, N° (%)**	9 (15)	13 (29)	6 (18)	0 (0)	0.058
**Fatigue**					
**FSS, median (IQR)**	23 (14 - 41)	26 (16 - 47)	28 (17 - 38)	13 (11 - 24)	0.235
**FSS > 36, N° (%)**	20 (33)	17 (38)	12 (35)	2 (20)	0.732
**Health-Related Quality of LIfe**					
**MCS, median (IQR)**	44 (30- 50)	36 (20 - 47)	35 (19 - 48)	41 (29 - 47)	0.216
**MCS < 50, N° (%)**	46 (77)	38 (84)	27 (79)	10 (100)	0.150
**PCS, median (IQR)**	51 (38 - 57)	47 (35 - 56)	42 (33 - 57)	57 (53 - 58)	**0.039**
**PCS < 50, N° (%)**	27 (45)	25 (56)	22 (65)	2 (20)	**0.047**
**Hospital Memories recall**					
**In-ICU Memories, N° (%)**	45 (75)	38 (84)	23 (67)	8 (80)	0.354

**Pre-ICU Memories, N° (%)**	36 (60)	29 (64)	19 (56)	5 (50)	0.797
**Delusional Memories, N° (%)**	50 (83)	30 (67)	20 (59)	4 (40)	**0.009**
**Factual Memories, , N° (%)**	50 (83)	39 (87)	31 (91)	10 (100)	0.258
**Feeling Memories, , N° (%)**	32 (53)	28 (62)	24 (70)	2 (20)	**0.026**

FSS: fatigue Severity Scale; HADS-A: Hospital Anxiety and Depression Scale-Anxiety; HADS-D: Hospital Anxiety and Depression Scale-Depression; ISI: Insomnia Severity Index; MCS: Mental Component Summary Measures of quality of life; PCL-5: PTSD Checklist for DSM-5; PCS: Physical Component Summary Measures of quality of life.

Table S3. Types of ICU memories for patients who retained ICU memories (n = 114).

	Patients with ICU memories (n = 114)
**Presence of Factual memories, N° (%)**	**99 (87)**
RELATIVES, n° (% of factual memories)	37 (37)
ALARMS	38 (38)
VOICES	60 (61)
LIGHTS	85 (86)
FACES	38 (38)
ENDOTRACHEAL TUBE	58 (59)
TRACHEAL ASPIRATIONS	47 (47)
DARKNESS	15 (15)
CLOCK	26 (26)
NASOGASTRIC TUBE	40 (40)
MEDICAL WARD	68 (69)
**Presence of Feeling memories, N° (%)**	**68 (60)**
DISCOMFORT n° (% of feeling memories)	33 (49)
DIZZINESS	29 (43)
DISCOURAGEMENT	22 (32)
ANXIETY	50 (74)
FEAR	19 (28)
PAIN	26 (38)
**Presence of delusional memories, N° (%)**	**79 (69)**
THREATENING n° (% of delusional	21 (27)
memories)	52 (66)
HALLUCINATIONS	46 (58)
NIGHTMARES	69 (87)
DREAMS	

Table S4. Patients characteristic according to the presence of Anxiety at six months from discharge. Anxiety is defined as an Hospital Anxiety and Depression Scale - Anxiety (HADS-A) score > 8.

	**Anxiety**<br>**N° = 30 (20%)**	**No Anxiety**<br>**N° = 119 (80%)**	**p**
**Age, years, median (IQR)**	58 (49-65)	61 (54-67)	0.371
**Sex, M, N°(%)**	18 (60)	91 (77)	0.057
**BMI. kg/m2m, median (IQR)**	29.9 (27.1-35.8)	28.9 (26.6-32.4)	0.110
**Charlson Index, median (IQR)**	0 (0-0)	0 (0-0)	0.751
**N of comorbidities. N°(%)**<br>**0**<br>**1**<br>**2**<br>**3**<br>**4**	 9 (31)<br>8 (28)<br>4 (14)<br>3 (10)<br>5 (17)	 32 (27)<br>36 (31)<br>21 (18)<br>14 (12)<br>15 (13)	 0.937
**SAPS II, median (IQR)**	40 (30-46)	35 (29-43)	0.192
**General Ward pre-ICU Length of stay, days, median, IQR**	3 (1-4)	3 (1-5)	0.875
**ICU Length Of Stay, days, median (IQR)**	18 (13-34)	17 (11-26)	0.329
**Hospital Length of Stay, days, median (IQR)**	47 (32-60)	40 (29-52)	0.172
**Duration of mechanical ventilation, days, median (IQR)**	14 (9-26)	13 (6-19)	0.258
**Tracheostomy, N°(%)**	5 (17)	25 (21)	0.623
**ECMO, N°(%)**	4 (13)	12 (10)	0.618
**Pronation, N°(%)**	25 (83)	94 (80)	0.651
**Corticosteroids, N°(%)**	21 (72)	80 (68)	0.776
**CRRT, N°(%)**	3 (10)	2 (2)	**0.022**
**Catecholamine, N°(%)**	6 (21)	18 (15)	0.490

BMI: Body Mass Index; CRRT: Continuous Renal Replacement Therapy; ECMO: Extracorporeal Membrane Oxygenation; ICU: Intensive Care Unit; MV: Mechanical; SAPSII: Simplified Acute Physiological Score II.

Table S5. Patients characteristic according to the presence of depression at six months from discharge. Depression is defined as an Hospital Anxiety and Depression Scale - Depression (HADS-D) score > 8.

	**Depression**<br>**N° = 33 (22.1%)**	**No Depression**<br>**N° = 116 (77.9%)**	**p**
**Age, years, median (IQR)**	59 (53-68)	61 (54-66)	0.932
**Sex, M, N°(%)**	19 (58)	90 (78.3)	**0.017**
**BMI. kg/m2m, median (IQR)**	31.3 (28-36)	28.7 (27-32)	0.075
**Charlson Index, median (IQR)**	0 (0-1)	0 (0-0)	0.221
**N of comorbidities. N°(%)**<br>**0**<br>**1**<br>**2**<br>**3**<br>**4**	 8 (25)<br>10 (31)<br>4 (13)<br>4 (13)<br>6 (19)	 33 (29)<br>34 (30)<br>21 (18)<br>13 (11)<br>14 (12)	0.833
**SAPS II, median (IQR)**	38 (32-45)	35 (29-43)	0.276
**General Ward pre-ICU Length of stay, days, median, IQR**	3 (1-5)	3 (1-5)	0.6133
**ICU Length Of Stay, days, median (IQR)**	19 (11-37)	17 (12-25)	0.364
**Hospital Length of Stay, days, median (IQR)**	45 (32-56)	39 (29-50)	0.141
**Duration of mechanical ventilation, days, median (IQR)**	13 (8-26)	13 (6-19)	0.219
**Tracheostomy, N°(%)**	6 (19)	24 (21)	0.776
**ECMO, N°(%)**	6 (18)	10 (9)	0.122
**Pronation, N°(%)**	26 (79)	93 (81)	0.791
**Corticosteroids, N°(%)**	26 (81)	75 (65)	0.184
**CRRT, N°(%)**	4 (13)	1 (1)	**0.001**
**Catecholamine, N°(%)**	7 (22)	17 (15)	0.348

BMI: Body Mass Index; CRRT: Continuous Renal Replacement Therapy; ECMO: Extracorporeal Membrane Oxygenation; ICU: Intensive Care Unit; MV: Mechanical; SAPSII: Simplified Acute Physiological Score II.

Table S6. Patients' characteristics according to the presence of Post Traumatic Stress Disorder (PTSD). PTSD is defined as a PTSD Checklist for DSM-5 (PCL-5) score >32.

	**PTSD**<br>**N° = 11 (7%)**	**no PTSD**<br>**N° = 138 (93%)**	**p**
**Age, years, median (IQR)**	57 (48-59)	61 (54-67)	0.073
**Sex, M, N°(%)**	6 (55)	103 (75)	0.135
**BMI. kg/m2m, median (IQR)**	31.3 (23.9-39.1)	29.1 26.7 32.5	0.537
**Charlson Index, median (IQR)**	0 (0-2)	0 0 0	0.668
**N of comorbidities. N°(%)**<br>**0**<br>**1**<br>**2**<br>**3**<br>**4**	 5 (46)<br>3 (27)<br>2 (18)<br>0 (0)<br>1 (9)	 36 (27)<br>41 (30)<br>23 (17)<br>17 (13)<br>20 (14)	0.574
**SAPS II, median (IQR)**	34 (29-52)	36 (29-43)	0.606
**General Ward pre-ICU Length of stay, days, median, IQR**	4 (1-7)	3 (1-5)	0.342
**ICU Length Of Stay, days, median (IQR)**	13 (5-21)	18 (12-28)	0.263
**Hospital Length of Stay, days, median (IQR)**	44 (13-48)	41 (29-52)	0.583
**Duration of mechanical ventilation, days, median (IQR)**	14 (5-21)	13 (7-20)	0.955
**Tracheostomy. n(%)**	1 (9)	29 (22)	0.328
**ECMO. n(%)**	2 (18)	14 (10)	0.413
**Pronation. n(%)**	10 (91)	109 (79)	0.362
**Corticosteroids. n(%)**	7 (64)	93 (68)	0.899
**CVVH. n(%)**	1 (9)	4 (3)	0.283
**catecholamine. n(%)**	2 (18)	22 (16)	0.871

BMI: Body Mass Index; CRRT: Continuous Renal Replacement Therapy; ECMO: Extracorporeal Membrane Oxygenation; ICU: Intensive Care Unit; MV: Mechanical; SAPSII: Simplified Acute Physiological Score II.

Table S7. Patients' characteristics according to the presence of Insomnia. Insomnia is defined as an Insomnia Severity Index (ISI) score >8.

	**Insomnia**<br>**N° = 28 (19%)**	**no Insomnia**<br>**N° = 121 (81)**	**p**
**Age, years, median (IQR)**	57 (48-62)	61 (55-67)	**0.031**
**Sex, M, N°(%)**	18 (67)	91 (75)	0.362
**BMI. kg/m2m, median (IQR)**	29.1 (26.9-33.1)	29.1 (26.6-32.8)	0.962
**Charlson Index, median (IQR)**	0 (0-0)	0 (0-0)	0.533
**N of comorbidities. N°(%)**			0.156
**0**	10 (37)	31 (26)	
**1**	9 (33)	35 (29)	
**2**	6 (22)	19 (16)	
**3**	2 (7)	15 (13)	
**4**	0 (0)	20 (17)	
**SAPS II, median (IQR)**	35 (29-47)	36 (29-43)	0.688
**General Ward pre-ICU Length of stay, days, median, IQR**	3 (1-6)	3 (1-5)	0.604
**ICU Length Of Stay, days, median (IQR)**	13 (8-23)	18 (12-28)	0.285
**Hospital Length of Stay, days, median (IQR)**	37 (25-50)	42 (30-52)	0.360
**Duration of mechanical ventilation, days, median (IQR)**	12 (8-17)	13 (6-21)	0.979
**Tracheostomy. n(%)**	2 (7)	28 (23)	0.061
**ECMO. n(%)**	3 (11)	13 (11)	0.956
**Pronation. n(%)**	24 (89)	95 (79)	0.219
**Corticosteroids. n(%)**	19 (70)	82 (68)	0.837
**CVVH. n(%)**	1 (4)	4 (3)	0.930
**catecholamine. n(%)**	4 (15)	20 (17)	0.801

BMI: Body Mass Index; CRRT: Continuous Renal Replacement Therapy; ECMO: Extracorporeal Membrane Oxygenation; ICU: Intensive Care Unit; MV: Mechanical; SAPSII: Simplified Acute Physiological Score II.

Table S8. Patients' characteristics according to the presence of Fatigue. Fatigue is defined as a Fatigue Severity Scale (FSS) score >36.

	**Fatigue**<br>**N° = 51 (34%)**	**no Fatigue**<br>**N° = 98 (66%)**	**p**
**Age, years, median (IQR)**	61 (57-69)	59 (51-66)	**0.027**
**Sex, M, N°(%)**	39 (77)	70 (72)	0.572
**BMI. kg/m2m, median (IQR)**	29.4 (26.8-35.8)	29.0 (26.6-32)	0.119
**Charlson Index, median (IQR)**	0 (0-1)	0 (0-0)	0.266
**N of comorbidities. N°(%)**<br>**0**<br>**1**<br>**2**<br>**3**<br>**4**	 16 (32)<br>15 (30)<br>7 (14)<br>5 (10)<br>7 (14)	 25 (26)<br>29 (30)<br>18 (19)<br>12 (12)<br>13 (13)	0.905
**SAPS II, median (IQR)**	38 (30-43)	35 (28-43)	0.355
**General Ward pre-ICU Length of stay, days, median, IQR**	3 (1-5)	3 (1-5)	0.903
**ICU Length Of Stay, days, median (IQR)**	20 (12-36)	16 (10-25)	0.084
**Hospital Length of Stay, days, median (IQR)**	46 (32-60)	39 (29-49)	**0.036**
**Duration of mechanical ventilation, days, median (IQR)**	12 (7-20)	13 (6-20)	0.971
**Tracheostomy. n(%)**	15 (30)	15 (16)	**0.041**
**ECMO. n(%)**	8 (16)	8 (8)	0.166
**Pronation. n(%)**	45 (88)	74 (76)	0.082
**Corticosteroids. n(%)**	40 (80)	61 (63)	0.074
**CVVH. n(%)**	3 (6)	2 (2)	0.217
**catecholamine. n(%)**	10 (20)	14 (15)	0.402

BMI: Body Mass Index; CRRT: Continuous Renal Replacement Therapy; ECMO: Extracorporeal Membrane Oxygenation; ICU: Intensive Care Unit; MV: Mechanical; SAPSII: Simplified Acute Physiological Score II.

Table S9. Patients characteristic according to the presence of below-threshold Physical Component Score (PCS) of Short Form Health Survey 36 (SF-36) at six months from discharge. Low PCS is defined as < 50.

	**Low PCS**<br>**N° = 76 (51%)**	**Normal PCS**<br>**N° = 73 (49%)**	**p**
**Age, years, median (IQR)**	61 (56-68)	57 (50-65)	**0.008**
**Sex, M, N°(%)**	54 (71)	55 (76)	0.461
**BMI. kg/m2m, median (IQR)**	29.9 (26.9-33.8)	28.5 (26.5-31.6)	**0.042**
**Charlson Index, median (IQR)**	0 (0-1)	0 (0-0)	0.144
**N of comorbidities. N°(%)**<br>**0**<br>**1**<br>**2**<br>**3**<br>**4**	 20 (27)<br>25 (33)<br>9 (12)<br>9 (12)<br>12 (16)	 21 (29)<br>19 (26)<br>16 (22)<br>8 (11)<br>8 (11)	0.463
**SAPS II, median (IQR)**	37 (30-43)	34 (27-43)	0.410
**General Ward pre-ICU Length of stay, days, median, IQR**	2 (0-5)	3 (1-5)	0.5230
**ICU Length Of Stay, days, median (IQR)**	18 (12-34)	17 (12-24)	0.183
**Hospital Length of Stay, days, median (IQR)**	45 (32-59)	38 (29-46)	**0.017**
**Duration of mechanical ventilation, days, median (IQR)**	12 (8-21)	13 (6-19)	0.374
**Tracheostomy, n(%)**	18 (24)	12 (17)	0.252
**ECMO, n(%)**	11 (15)	5 (7)	0.140
**Pronation, n(%)**	66 (87)	53 (74)	**0.043**
**Corticosteroids, n(%)**	58 (77)	43 (60)	**0.021**
**CVVH, n(%)**	4 (5)	1 (1)	0.270
**catecholamine, n(%)**	11 (15)		0.603

BMI: Body Mass Index; CRRT: Continuous Renal Replacement Therapy; ECMO: Extracorporeal Membrane Oxygenation; ICU: Intensive Care Unit; MV: Mechanical; SAPSII: Simplified Acute Physiological Score II.

Table S10. Patients characteristic according to the presence of below-threshold Mental Component Score (MCS) of Short Form Health Survey 36 (SF-36) at six months from discharge. Low MCS is defined as < 50.

	**Low MCS**<br>**N° = 121 (81.2)**	**Normal MCS**<br>**N° = 28 (18.8)**	**p**
**Age, years, median (IQR)**	60 (52-67)	58 (55-65)	0.447
**Sex, M, N°(%)**	90 (75)	19 (68)	0.440
**BMI. kg/m2m, median (IQR)**	29,1 (26,8-33,2)	28,1 (25.3-31.7)	0.199
**Charlson Index, median (IQR)**	0 (0-0)	0 (0-0)	0.528
**N of comorbidities. N°(%)**<br>**0**<br>**1**<br>**2**<br>**3**<br>**4**	 36 (30)<br>36 (30)<br>18 (15)<br>12 (10)<br>17 (14)	 5 (18)<br>8 (29)<br>7 (25)<br>5 (18)<br>3 (11)	0.411
**SAPS II, median (IQR)**	35 (29-43)	38 (27-43)	0.465
**General Ward pre-ICU Length of stay, days, median, IQR**	3 (1-5)	3 (0-5)	0.513
**ICU Length Of Stay, days, median (IQR)**	17 (11-28)	18 (12-25)	0.778
**Hospital Length of Stay, days, median (IQR)**	40 (29-52)	45 (36-57)	0.485
**Duration of mechanical ventilation, days, median (IQR)**	12 (6-21)	15 (9-19)	0.290
**Tracheostomy, n(%)**	26 (22)	4 (15)	0.414
**ECMO, n(%)**	10 (8)	6 (21)	**0.044**
**Pronation, n(%)**	100 (83)	19 (68)	0.063
**Corticosteroids, n(%)**	80 (67)	21 (75)	0.425
**CVVH, n(%)**	5 (4)	0 (0)	0.278
**catecholamine, n(%)**	21 (18)	3 (11)	0.408

BMI: Body Mass Index; CRRT: Continuous Renal Replacement Therapy; ECMO: Extracorporeal Membrane Oxygenation; ICU: Intensive Care Unit; MV: Mechanical; SAPSII: Simplified Acute Physiological Score II.

Table S11. Short Form Health Survey 36 (SF-36) results according to the presence of pre-ICU and ICU memories.

	All patients (N° =149)	Pre-ICU memories (N° = 89)	No pre-ICU memories (N° =60)	p	in-ICU memories (N° = 114)	No in-ICU memories (N° = 35)	p
36-Item Short Form Health Survey 36 (SF-36)							
**SF-36 Physical Functioning. median. IQR**	80<br>55 - 95	85<br>65 - 95	67.5<br>37.5 - 90	**0.001**	85<br>55 - 95	65<br>35 - 90	**0.025**
**SF-36 Role Function - Physical. median. IQR**	50<br>0 - 100	100<br>25 - 100	37.5<br>0 - 100	**0.004**	75<br>25 - 100	25<br>0 - 100	**0.011**
**SF-36 Role Function - Emotional. median. IQR**	100<br>33.3 - 100	100<br>66.7 - 100	33.3<br>0 - 100	**<0.001**	100<br>33.3 - 100	66.7<br>0 - 100	0.221
**SF-36 Vitality. median. IQR**	60<br>50 - 75	65<br>60 - 80	55<br>40 - 65	**<0.001**	65<br>50 - 80	55<br>45 - 65	**0.033**
**SF-36 Social Functioning. median. IQR**	75<br>62.5 - 90	87.5<br>67.5 - 100	63.75<br>50 - 87.5	**<0.001**	75<br>62.5 - 100	75<br>50 - 87.5	**0.027**
**SF-36 Emotional Well Being. median. IQR**	72<br>60 - 88	76<br>68 - 88	68<br>52 - 80	**0.001**	74<br>60 - 88	72<br>60 - 84	0.488
**SF-36 Pain median IQR**	77.5<br>55 - 100	90<br>67.5 - 100	67.5<br>45 - 90	**0.000**	80<br>57.5 - 100	67.5<br>45 - 100	**0.017**
**0SF-36 General Health. median. IQR**	65<br>45 - 80	70<br>55 - 80	55<br>40 - 75	**0.004**	65<br>50 - 80	60<br>35 - 75	0.095

ICU: Intensive Care Unit.

Table S12. Multivariable analysis.

**Anxiety (HADS-A > 7)**	**OR**	**CI 95%**	**p**
Presence of Pre-ICU memories	0.253	0.108 - 0.592	**0.002**
**Depression (HADS-D > 7)**	**OR**	**CI 95%**	**p**
Presence of Pre-ICU memories	0.148	0.053 - 0.417	**< 0.001**
CRRT	26.203	2.268 - 302.7	**0.009**
Gender (male)	0.245	0.089 - 0.676	**0.007**
Prone position	0.228	0.067 - 0.773	**0.018**
BMI	1.101	1.008 - 1.202	**0.032**
Pre ICU days	1.158	1.009 - 1.329	**0.036**
**PTSD (PCL-5 > 32)**	**OR**	**CI 95%**	**p**
Gender (male)	0.233	0.058 - 0.939	**0.040**
Pre ICU days	1.159	1.003 - 1.338	**0.045**
Feeling memories	8.524	1.035 - 70.215	**0.046**
**Insomnia (ISI > 8)**	**OR**	**CI 95%**	**p**
-	-	-	-
**Fatigue (FSS > 36)**	**OR**	**CI 95%**	**p**
Presence of Pre-ICU memories	0.469	0.219 - 1.001	**0.050**
Corticosteroids	2.814	1.180 - 6.709	**0.020**
BMI	1.113	1.028 - 1.205	**0.008**
age	1.057	1.013 - 1.104	**0.010**
**Low PCS (PCS < 50)**	**OR**	**CI 95%**	**p**
BMI	1.126	1.035 - 1.225	**0.006**
Hospital length of stay	1.036	1.014 - 1.059	**0.001**
Age	1.056	1.013 - 1.100	**0.010**
Steroids	4.737	1.914 - 11.722	**0.001**
Catecholamines	0.244	0.068 - 0.880	**0.031**
Presence of post ICU memories	0.391	0.174 - 0.879	**0.023**
**Low MCS (MCS < 50)**	**OR**	**CI 95%**	**p**
ECMO	0.146	0.347 - 0.611	**0.008**
Pre ICU days	1.207	1.018 - 1.431	**0.030**

Presence of ICU memories	0.136	0.024 - 0.767	**0.024**
ICU LOS	1.087	1.014 - 1.165	**0.019**
Hospital LOS	0.948	0.906 - 0.992	**0.020**

BMI: Body Mass Index; CRRT: Continuous Renal Replacement Therapy; ECMO: Extracorporeal Membrane Oxygenation; FSS: fatigue Severity Scale; HADS-A: Hospital Anxiety and Depression Scale-Anxiety; HADS-D: Hospital Anxiety and Depression Scale-Depression; ICU: Intensive Care Unit; ISI: Insomnia Severity Index; LOS: Length of Stay; MCS: Mental Component Summary Measures of quality of life; PCL-5: PTSD Checklist for DSM-5; PCS: Physical Component Summary Measures of quality of life
